# *CCND1* gene polymorphic variants in patients with differentiated thyroid carcinoma

**DOI:** 10.3892/ol.2014.2617

**Published:** 2014-10-15

**Authors:** SZYMON HRYHOROWICZ, KATARZYNA ZIEMNICKA, MARTA KACZMAREK-RYŚ, JUSTYNA HOPPE-GOŁĘBIEWSKA, ANDRZEJ PŁAWSKI, MARZENA SKRZYPCZAK-ZIELIŃSKA, MAŁGORZATA SZKUDLAREK, MONIKA GOŁĄB, BARTŁOMIEJ BUDNY, MAREK RUCHAŁA, RYSZARD SŁOMSKI

**Affiliations:** 1 NanoBioMedical Center, Adam Mickiewicz University, Poznań 61-614, Poland; 2 Department of Endocrinology, Metabolism and Internal Diseases, Poznan University of Medical Sciences, Poznań 60-355, Poland; 3 Institute of Human Genetics, Polish Academy of Sciences, Poznań 60-479, Poland; 4 Department of Biochemistry and Biotechnology, University of Life Sciences, Poznań 60-632, Poland

**Keywords:** differentiated thyroid carcinoma, papillary thyroid carcinoma, *CCND1* gene, cancer susceptibility alleles with low penetrance

## Abstract

Alterations in the *CCND1* gene affect the cell cycle and are frequently observed in a variety of cancers. While the most frequent mutations that occur in thyroid tumor tissue have been characterized, the genetic factors that predispose individuals to differentiated thyroid cancer (DTC) remain to be elucidated. The present study examined whether the *CCND1* c.723G>A (rs9344; p.Pro241=) and c.669C>T (rs3862792; p.Phe223=) variants have an impact on DTC susceptibility. A cohort consisting of 652 patients diagnosed with DTC were analyzed and comapred with a reference group of 799 subjects from the general population. Pyrosequencing was used as the genotyping technique. In order to determine the statistical significance of differences observed in the genotypic and allelic frequencies between the compared groups, GraphPad Prism 4 was used. At the rs9344 locus in the DTC patients, a higher frequency of allele A [P=0.032; odds ratio (OR), 1.18; 95% confidence interval (CI), 1.014–1.361] and the AA homozygous genotype (P=0.028; OR, 1.41; 95% CI, 1.059–1.989) was observed compared with the control population group. The differences were stronger for papillary carcinomas (OR 1.45; 95% CI, 1.059–1.989), but were not significant in follicular tumors. No statistically significant differences were noted in the frequency of genotypes or alleles at the rs3862792 locus in the examined groups. The present findings indicate that the c.723A variant of the *CCDN1* gene may be a susceptibility low penetrance allele in the development of papillary thyroid cancer in the population studied, however it does not impact on multifocality, metastatic ability or age at diagnosis. A cumulative effect of the analyzed *CCND1* gene variants was also excluded.

## Introduction

Differentiated thyroid carcinoma (DTC) accounts for ~1.5% of all malignancies in the Polish population, and its incidence is increasing worldwide. This may be due to evolving diagnostic technology and medical surveillance practices, however, the technological advances in medical sciences only partly explain the observed rise in this incidence. The genesis of thyroid cancer is complex and connected with several factors, including exposure to chemical agents or irradiation, iodine deficiency and genetic predisposition (1).

Epidemiological data reports >1,700 new cases of DTC in Poland each year (2). Depending on the population, thyroid cancer occurs 4–6 times less in males compared with females. This ratio indicates a different molecular background of thyroid cancer in the two genders (3). According to the histopathology of DTC, mainly papillary thyroid carcinoma (PTC) and follicular thyroid carcinoma (FTC) can be distinguished. Hürthle cell or oxyphilic malignant tumors are infrequently detected. DTC is a slowly developing tumor that is often asymptomatic and constitutes a major problem for appropriate advanced detection.

The majority of DTCs are of a relatively low grade, but metastases to adjacent and distant tissues are extremely common and include lung, bone and brain metastases (4–6). For effective treatment, consisting of total thyroidectomy connected with radioiodine ^131^I therapy, early diagnosis of the disease is essential (7,8). Worldwide, studies are investigating the genetic predictors of malignancies. Polymorphisms in genes critical to cell cycle control are strong candidates for an elevated risk of cancer.

Cell cycle kinases play a major role in thyroid carcinoma metastasis. The cell cycle is controlled by cyclin-dependent kinases (CDK), which are activated by the formation of complexes with cyclins. The *CCND1* gene, located on chromosome 11q23, encodes a positive regulator of the cell cycle, a nuclear protein that forms complexes with CDKs 4 and 6, and phosphorylates and inactivates the retinoblastoma protein, which is involved in the transition from the G_1_ to S phase (9).

The relevance of the cyclin D1 gene in cancer was apparent upon its identification; this locus had been indicated as involved in a chromosomal rearrangement of benign parathyroid tumors (10). Mutations in the *CCND1* gene that altered cell cycle progression have been frequently observed in various tumors and may contribute to tumorigenesis. Germline sequence variants of this gene have been the subject of numerous studies into cancer diseases. The evidence indicates that common single nucleotide polymorphisms (SNPs), which are known to alter the splicing of the cyclin D1 transcript, may modify the action of cyclin D1 in cancer cells and affect the cancer risk and outcome (11).

The aim of the present study was to determine if the *CCND1* gene polymorphisms, c.723G>A (rs9344; p.Pro241=) and c.669C>T (rs3862792; p.Phe223=), have an impact on the occurrence of FTC and PTC. The present study also aimed to demonstrate an association between the aforementioned SNP allelic variants and DTC, with particular reference to gender, histological subtype, patient age at the time of diagnosis, tumor-node-metastasis staging and the occurrence of metastases.

## Materials and methods

### Groups

A group of 647 patients with DTC (567 females and 80 males) from Wielkopolska (Greater Poland) was examined. The gender ratio (female:male) was 7.1:1, which varies slightly from previously reported gender ratios (1). Among the examined samples, 565 were obtained from patients diagnosed with papillary thyroid carcinoma. In the PTC patients, the gender ratio was 7.8:1. The investigated group also included 64 cases diagnosed with FTC. The gender ratio for the FTC patients was 5.9:1. The present study focused on two main histological types of DTCs, papillary and follicular. To obtain general statistics for DTC patients, 18 patients diagnosed with oxyphilic thyroid cancer, carcinoma insulare and Hürthle cell tumors were included. Furthermore, 45 patients with nodular goiter were included as a control group. The nodular goiter group was selected on the basis of a cancer-negative histopathological examination performed following a total thyroidectomy. Due to the low numbers, the nodular goiter group was considered only in a comparison with the similarly numbered FTC group.

As a comparative group, 799 individuals from the general population, 615 females and 184 males, were enrolled in the present study. The number of individuals in the female group was increased three times to align with the DTC patient female group.

All patients with thyroid cancer and nodular goiter were examined and hospitalized in the Department of Endocrinology, Metabolism and Internal Diseases of Poznań University of Medical Sciences (Poznań, Poland). The population group consisted of unrelated individuals from the Wielkopolska region who attended the hospital for paternity testing.

All individuals examined in the present study agreed to take part in the genetic testing, in accordance with the requirements of the Ethical Committee of Poznań University of Medical Science (acceptance no. 629/07).

### Genotyping

DNA was obtained from whole blood leukocytes by guanidine thiocyanate and subsequent phenol-chloroform extraction. Pyrosequencing was used as the genotyping technique.

The sequence of the primers used in the analysis were designed by PyroMark Assay Design Software 2.0 (Qiagen, Venlo, Limburg, Netherlands). The primer sequences used in the analysis of the *CCND1* gene were as follows: c.723G>A (rs9344) forward, biotinylated 5′-TCCTACTACCGCCTCACACGC and reverse, 5′-GCACTAGGTGTCTCCCCCTGTAA (amplicon length, 112 bp; temperature, 67°C), and sequencing primer, 5′-GGACATCACCCTCACTTA; and c.669C>T (rs3862792) forward, 5′-AATCCGCCCTCCATGGTG and reverse, biotinylated 5′-CGTGTGAGGCGGTAGTAGGAC (amplicon length, 101 bp; temperature, 65°C), and sequencing primer, 5′-AACCTGAGGAGCCCC. The pyrosequencing reactions were performed on a PSQ96 device (Pyrosequencing AB, Uppsala, Sweden) using the PyroMark Gold Q96 reagents kit (Qiagen).

### Statistical analysis

The prevalence of the genotypes in the patients and control individuals was examined by the Hardy-Weinberg equilibrium (12). The χ^2^ test was used to evaluate the differences in genotypic and allelic frequencies between the groups. Furthermore, statistical analyses that assessed the gender, histological type of the cancer, patient age at diagnosis and metastatic potential were performed.

In order to determine the statistical significance of the differences observed between the compared groups, GraphPad Prism 4 (GraphPad Software, Inc., La Jolla, CA, USA) was used to obtain odds ratios (OR), 95% confidence intervals (CI) and P-values. P<0.05 was considered to indicate a statistically significant difference.

The linkage disequilibrium between the c.723G>A (rs9344) and c.669C>T (rs3862792) polymorphisms of the *CCND1* gene was analyzed by Haploview v.3.11 (Broad Institute, Cambridge, MA, USA) (13).

## Results

### Geneotyping

**Figure 1:**
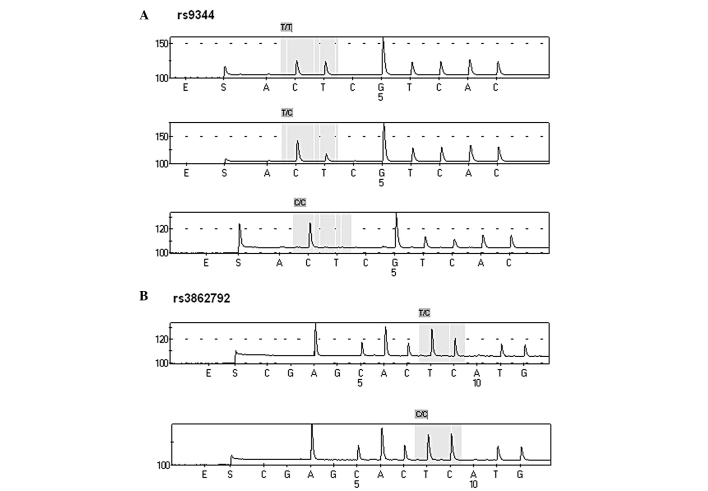
Pyrograms obtained from the analysis of *CCND1* gene polyporphisms. Polymorphic sites are marked with gray shading. (A) Pyrograms for the three genotypes obtained from the analysis of the *CCND1* gene variant c.723G>A (rs9344). (B) Pyrograms for the two genotypes obtained from the analysis of *CCND1* gene variant c.669C>T (rs3862792).

The genotyping results of the c.723G>A (rs9344) locus were obtained by pyrosequencing (Fig. 1) for all the 647 patients with DTC and the 799 individuals from the population. Following comparison with the dbSNP database (14), the results obtained for the population group were found to be similar to the results for other European populations. The ancestral G allele was more frequent than the A allele.

The difference in the frequency of the alleles and genotypes that was noted in the patients with thyroid cancer appeared to be even more important (Table I). A significantly higher frequency of the minor A allele was observed in patients with DTC, but the OR remained near 1. When the AA genotype frequency was compared between the two groups, a statistically significant difference was obtained. Comparison between AA homozygotes and GA heterozygotes revealed a noticeable and statistically significant difference. It was concluded that variant c.723A in homozygous and heterozygous configurations is a risk factor for DTC, but that it exhibits low penetrance.

Due to an almost seven-fold higher incidence of thyroid cancer in females, statistical analysis was performed separately for each gender. By comparing the females with DTC against those from the population group, an association between genotype AA and thyroid cancer was identified, but no relevant associations in the male patients were noted and there were no statistically significant differences between the genders.

### Association study

In the next step, the histological type of thyroid tumor was focused upon. PTC was observed almost nine times more frequently than FTC in patients with thyroid cancer. Statistical calculations revealed that the minor A allele was present significantly more in the PTC patients and that the association observed for homozygous AA was even more apparent. When females with PTC were compared with the whole population, the statistically significant difference remained. No statistically significant differences were observed for the alleles and genotype frequencies between the patients diagnosed with FTC and the population or control group with nodular goiter.

In the analysis of c.669C>T (rs3862792), pyrosequencing results were obtained for 642 DTC patients and 628 individuals from the population.

No differences in allele and genotype frequencies were observed. Additionally, the rare TT genotype was absent in the whole analyzed group (Table II). A comparison between the patient gender and histological type of thyroid tumor did not reveal any significant differences. In conclusion, variant c.669C>T is not a contributing factor in DTC development.

Statistical tests were also performed to identify possible correlations between the carried genotype and the age at diagnosis. In the designed age groups, any correlation between the variables in results obtained for the rs9344 and rs3862792 locus analysis were noted (Table III).

Metastases and recurrences were present in 123 DTC patients (19%). In the patient group, the main metastases observed were to the cervical and mediastinal lymph nodes, lungs and bones. No significant association between the occurrence of metastases and the alleles or genotypes of the two polymorphic sequence variants was noted. There was also no significant correlation between the studied SNP variant presence and tumor size (Table IV). However, in T1-stage patients, the presence of the risk allele, c.723A, was noted to occur with considerably lower frequency, but this group consisted of only 12 individuals with DTC, which made this observation questionable.

### Haplotype analysis

The analysis of haplotypes formed by polymorphisms located in close proximity to each other allows for the detection of associations that are invisible in the analysis of single polymorphisms. Therefore, an analysis of linkage disequilibrium was performed, comparing the two polymorphisms of the *CCND1* gene. This analysis was performed using Haploview v.3.11 (Broad Institute) (13).

The distance between the c.669C>T and c.723G>A polymorphisms is only 54 bp. The two polymorphisms demonstrate extremely strong linkage disequilibrium, with a D′ value of 0.84–1.0, although the correlation coefficient between these two loci does not allow for the clear identification of a single allele of one polymorphism based on information from the second SNP allele (r^2^=0.27). The strong linkage disequilibrium enabled the haplotypes for 542 DTC patients and 512 individuals from the population to be determined.

The polymorphisms formed three haplotypes, CG, CA and TA, which were present in the DTC patients with a frequency of 51.7, 45.6 and 2.6%, respectively, and in the population with a frequency of 56.9, 40.3 and 2.7%, respectively. The CA haplotype was 5.3% more frequent in the DTC group. The risk allele, c.723A, was present in haplotypes with allele c.669T and c.669C.

## Discussion

Numerous variations of low-penetrance genes affecting a high proportion of the general population are likely to control DTC susceptibility (14). According to the latest published data concerning low-risk cancer susceptibility alleles, the present study examined two *CCND1* gene polymorphisms in a cohort of patients with DTC and in the general Polish population (15,16).

The present results indicate that variant c.723A may be a risk allele in the development of DTC in the Polish population, particularly in homozygotes. Statistical analyses regarding patient gender and the histological type of the tumor decreased the relevant significant associations to a group of females with PTC. These results confirmed that PTC and FTC are distinct, and that the molecular background of thyroid cancer in females and males may depend on different genetic factors.

Analysis of the rs3862792 variant excluded its association with DTC. The present study also revealed a lack of correlation between the subjected SNP alleles and the age at diagnosis, metastasis and multifocality.

Mutations, amplification and overexpression of the *CCND1* gene, which alter cell cycle progression, are observed frequently in a variety of tumors and may contribute to tumorigenesis. The germline sequence variants of this gene have been investigated by numerous studies of various cancers, and have indicated that the common polymorphism that alters splicing of the cyclin D1 transcript may also have an effect on the cancer risk and outcome (10). Among the numerous variants of the *CCND1* gene reported in databases, variant c.723G>A (rs9344; p.Pro241=) in exon 4 is one of the most frequently cited in cancer research. This polymorphism increases the frequency of the alternate splicing of cyclin D1 mRNA; allele c.723A results in a higher level of transcripts encoding a protein with an altered C-terminal domain. This variant may have a longer half-life than the c.723G variant, which may cause the G^1^/S-checkpoint bypass (11). Published data concerning the association between the c.723G>A polymorphism and the neoplastic process remains inconclusive, although several meta-analyses postulate the presence of a correlation between the c.723A allele and various types of cancers, including breast, gastric, bladder and cervical cancer or squamous cell carcinoma of the head and neck (17–20). Absenger *et al* revealed that, in the validation set, the c.723A allele remained significantly associated with a decreased time to tumor recurrence in univariate and multivariate analyses in patients with colon cancer. The study hypothesized that this polymorphism may be a prognostic biomarker in stage T2/T3 colon cancer patients and may be useful in predicting the clinical outcome of colon cancer patients (21).

The frequency of the c.723G>A polymorphism in Caucasians is 44–47% for the minor allele, c.723A, and 54–56% for the ancestral allele, c.723G, depending on the study (21,22), but large variances between racial and ethnic groups have been reported. The present results obtained from the Polish population are consistent with the aforementioned data.

The c.669C>T variant (rs3862792; p.Phe223=) is located in exon 3 and is a rare polymorphism. This polymorphism has been investigated in studies on ovarian, uterine, cervical (23,24) and breast cancer susceptibility alleles in European Caucasians (25,26), however no clear correlations have been identified. Studies into the c.669C>T variant in colorectal cancer in the UK population led to the conclusion that rare variants of the *CCND1* gene are risk factors for colorectal cancer, with considerably larger effects compared with common polymorphisms, and as such should be systematically evaluated in susceptibility studies (27,28). The frequency of the c.669C allele in Caucasians is ~96% and the frequency of the minor allele, c.669T, is 4% (29). Differences between various populations were noticed, although in the present study using the Polish population, similar frequencies were found to those reported in databases. In the present study, the c.669C>T SNP did not correspond with DTC.

The present results suggested that the c.723A variant may be a risk factor for DTC occurrence in homozygotes. The present population-based, retrospective study found that the c.723A variant is associated with an increased risk of PTC.

The present findings would require confirmation through large, population-based, unbiased studies of other ethnic groups.

## Figures and Tables

**Table I:** Frequency of alleles and genotypes of the *CCND1* gene variant, c.723G>A (rs9344), in DTC patients and the general population.

		Genotypes, n (%)					Alleles, n (%)			
							
Group	Total, n (%)	GG	GA	AA	P-value (GG vs. AA)	OR (95%CI)	G	A	P-value	OR (95% CI)
DTC patients	647 (100.00)	160 (24.73)	344 (53.17)	143 (22.10)	-	-	664 (51.31)	630 (48.69)	-	-
PTC	565 (87.33)	141 (24.96)	294 (52.04)	130 (23.01)	0.02023^a^	1.452 (1.059–1.989)^a^	576 (50.97)	554 (49.03)	0.02498^a^	1.191 (1.022–1.387)^a^
FTC	64 (9.89)	15 (23.44)	39 (60.94)	10 (15.63)	0.90866	1.050 (0.459–2.398)	69 (53.91)	59 (46.09)	0.75709	1.059 (0.738–1.519)
Remaining	18 (2.78)	4 (22.22)	11 (61.11)	3 (16.67)	-	-	19 (52.78)	17 (47.23)	-	-
DTC females	567 (87.63)	142 (25.04)	299 (52.73)	126 (22.22)	0.02706^a^	1.428 (1.041–1.960)^a^	583 (51.41)	551 (48.59)	0.03072^a^	1.183 (1.016–1.378)^a^
DTC males	80 (12.36)	18 (22.50)	45 (56.25)	17 (21.25)	0.29163	1.522 (0.696–3.328)	81 (50.63)	79 (49.38)	0.25640	1.240 (0.855–1.799)
Nodular goiter	45 (100.00)	11 (24.45)	28 (62.22)	6 (13.33)	0.76866	0.859 (0.311–2.371)	50 (55.56)	40 (44.44)	0.96697	0.991 (0.646–1.519)
Whole population	799 (100.00)	233 (29.16)	418 (52.32)	148 (18.52)	-	-	884 (55.32)	714 (44.68)	-	-
Females	615 (76.97)	175 (28.46)	328 (53.33)	112 (18.21)	-	-	678 (55.12)	552 (44.88)		
Males	184 (23.02)	58 (31.52)	90 (48.91)	36 (19.57)	-	-	206 (55.98)	162 (44.02)	-	-

a Significant at 95% CI.

OR, odds ratio; CI, confidence interval; DTC, differentiated thyroid carcinoma; PTC, papillary thyroid carcinoma; FTC, follicular thyroid carcinoma.

**Table II:** Frequency of alleles and genotypes of the *CCND1* gene variant, c.669C>T (rs3862792), in DTC patients and the general population.

		Genotypes, n (%)					Alleles, n (%)			
							
	Total, n (%)	CC	CT	TT	P-value (CC vs. CT)	OR (95% CI)	C	T	P-value	OR (95% CI)
DTC whole group	642 (100.00)	608 (94.70)	34 (5.30)	NO	0.87219	1.042 (0.634–1.710)	1250 (97.35)	34 (2.65)	0.87390	1.040 (0.638–1.697)
PTC	561 (87.38)	531 (94.65)	30 (5.35)	NO	0.89109	1.037 (0.619–1.737)	1092 (97.33)	30 (2.67)	0.89254	1.036 (0.622–1.723)
FTC	62 (9.66)	59 (95.16)	3 (4.84)	NO	0.78719	0.847 (0.252–2.840)	121 (97.58)	3 (2.42)	1.00790	0.850 (0.257–2.813)
Remaining	19 (2.96)	18 (94.74)	1 (5.26)	NO	-	-	37 (97.37)	1 (2.63)	-	-
DTC females	563 (100.00)	534 (94.85)	29 (5.15)	NO	-	-	1097 (97.42)	29 (2.58)	-	-
DTC males	79 (100.00)	74 (93.67)	5 (6.33)	NO	-	-	153 (96.84)	5 (3.16)	-	-
Nodular goiter	45 (100.00)	45 (100.00)	NO	NO	-	-	45 (100.00)	NO	-	-
Whole population	628 (100.00)	596 (94.90)	32 (5.10)	NO	-	-	1224 (97.45)	32 (2.55)	-	-
Females	427 (67.99)	404 (94.61)	23 (5.39)	NO	-	-	831 (97.31)	23 (2.69)	-	-
Males	201 (32.01)	192 (95.52)	9 (4.48)	NO	-	-	393 (97.76)	9 (2.24)	-	-

NO, not observed; OR, odds ratio; CI, confidence interval; DTC, differentiated thyroid carcinoma; PTC, papillary thyroid carcinoma; FTC, follicular thyroid carcinoma.

**Table III:** Frequency of alleles and genotypes of the *CCND1* gene variant, c.723G>A (rs9344), in differentiated thyroid carcinoma patients following division into age groups. The age at diagnosis was available for 514 patients.

Age group, years	Number of patients	Allele c.723G frequency, n (%)	Allele c.723A frequency, n (%)	OR	CI	P-value
≤20	15	16 (53.33)	14 (46.67)	0.892	0.431–1.848	0.76
21–40	152	160 (52.63)	144 (47.37)	0.895	0.685–1.169	0.42
41–60	243	245 (50.41)	241 (49.59)	1.112	0.874–1.413	0.39
≥61	114	109 (47.81)	119 (52.19)	1.152	0.859–1.545	0.35

OR, odds ratio; CI, confidence interval.

**Table IV:** Frequency of alleles and genotypes the *CCND1* gene variant c.723G>A (rs9344) in differentiated thyroid carcinoma patients with regard to tumor-node-metastasis staging.

	Staging													
	
Factor	T1		T1a		T1b		T2		T3		T4		Tx	
Number of patients, n (%)	12 (2.46)		180 (36.97)		41 (8.42)		93 (19.10)		66 (13.55)		68 (13.96)		27 (5.54)	
c.723G>A														
Allele	G	A	G	A	G	A	G	A	G	A	G	A	G	A
Frequency, %	70.8	29.2	46.4	53.6	41.5	58.5	46.2	53.8	50.0	50.0	53.7	46.3	37.0	63.0
c.723A positivity														
OR	0.226		0.989		1.108		1.160		1.172		0.837		1.829	
CI	0.070–0.725		0.641–1.524		0.513–2.396		0.672–2.002		0.624–2.204		0.466–1.501		0.619–5.402	
P-value	0.007		0.959		0.793		0.595		0.621		0.550		0.268	
c.669C>T														
Allele	C	T	C	T	C	T	C	T	C	T	C	T	C	T
Frequency, %	95.8	4.2	96.1	3.9	98.8	1.2	96.8	3.2	97.5	2.5	2.2	97.8	96.3	3.7
c.669T positivity														
OR	1.398		1.534		0.359		1.063		0.695		0.670		1.234	
CI	0.174–11.206		0.730–3.222		0.048–2.709		0.422–2.680		0.205–2.359		0.198–2.273		0.278–5.477	
P-value	0.751		0.256		0.300		0.897		0.557		0.518		0.782	

T, tumor; OR, odds ratio; CI, confidence interval.
